# Implementation einer Sturzsensorik in der Langzeitpflege: Eine Interviewstudie zur Betrachtung fördernder und hemmender Faktoren aus Sicht professionell Pflegender

**DOI:** 10.1007/s00391-023-02255-3

**Published:** 2023-11-08

**Authors:** Marie-Christin Redlich, Florian Fischer

**Affiliations:** Bayerisches Zentrum Pflege Digital, Hochschule für angewandte Wissenschaften Kempten, Albert-Einstein-Str. 6, 87437 Kempten, Deutschland

**Keywords:** Altenpflege, Stationäre Langzeitversorgung, Digitalisierung, Sturzerkennung, Sturzprävention, Elderly care, Long-term inpatient care, Digitalization, Fall detection, Fall prevention

## Abstract

**Hintergrund und Ziel:**

Digitale Unterstützungssysteme erlangen in der stationären Langzeitversorgung zunehmend Bedeutung. Die Technologien haben einerseits das Potenzial, einen wertvollen Beitrag zur Aufrechterhaltung der Selbstständigkeit im fortgeschrittenen Alter zu leisten. Andererseits können sie die Arbeitsprozesse professionell Pflegender unterstützen. Das Ziel der Studie war es, die Erwartungen an und Erfahrungen mit einer neuen Technologie – am Beispiel einer Sturzsensorik – aus Sicht professionell Pflegender zu erheben.

**Methode:**

Es wurde ein qualitatives Design verwendet, bei dem teilstrukturierte Interviews in 2 langzeitstationären Einrichtungen erfolgten. In einer Einrichtung fanden 3 Einzelinterviews und in der anderen langzeitstationären Pflegeeinrichtung ein Gruppeninterview mit 3 Pflegefachkräften statt. Zusätzlich erfolgte in jeder Einrichtung ein Einzelinterview mit einer Person in Leitungsfunktion. Zwischen der Implementation der Sturzsensoren und den Interviews lag ein Zeitrahmen von einem bis 3 Monaten. Die Daten wurden mittels qualitativer Inhaltsanalyse in MAXQDA ausgewertet.

**Ergebnisse und Diskussion:**

Grundsätzlich konnte durch die Studie aufgezeigt werden, dass eine Übereinstimmung zwischen Erwartungen an und retrospektiven Erfahrungen mit der neuen Technologie bei den professionell Pflegenden bestand. Die Studie ergab folgende fördernde Faktoren der Implementation: die zeitnahe Information über die Sturzgefährdung oder einen erfolgten Sturz, die Aufrechterhaltung der Autonomie und Bewegungsfreiheit der Bewohner:innen sowie die Verbesserung des Sicherheitsgefühls der Pflegefachkräfte und den damit im Zusammenhang stehenden psychischen Entlastungseffekt. Als hemmende Faktoren der Implementation standen erforderliche Voreinstellungen, Fehlalarme sowie der fehlerhafte Umgang mit der Technologie aufgrund von nicht vorhandenem Wissen im Vordergrund.

**Zusatzmaterial online:**

Zusätzliche Informationen sind in der Online-Version dieses Artikels (10.1007/s00391-023-02255-3) enthalten.

## Abstract (en)

**Background and objective:**

Digital support systems are becoming increasingly more important in long-term inpatient care facilities. Welfare technologies have the potential to make a valuable contribution to maintaining independence in advanced age. At the same time the technologies can support professional caregivers. The aim of the study was to describe the expectations and experiences with a new technology, using the example of a fall sensor system, from the perspective of professional caregivers.

**Method:**

We used a qualitative design with semistructured interviews in two long-term inpatient care facilities. In one facility, three individual interviews took place, while in the other long-term care facility, a group interview with three nursing professionals was conducted. Additionally, one individual interview was conducted with a person in a leadership role in each facility. The time from implementation of the fall sensors to the interviews was between 1 and 3 months. Data were analyzed using qualitative content analysis in MAXQDA.

**Results and discussion:**

The study demonstrated that there was a correspondence between expectations of and retrospective experiences with the new technology among professional nurses. The main facilitating factors of the implementation that were identified were timely information about the risk of falling or a fall that has occurred, maintenance of residents’ autonomy and freedom of movement as well as the enhancement of the sense of security among nursing professionals and the associated psychological relief effect. The inhibiting factors of the implementation were compulsory presetting, false alarms and faulty handling of the technology due to missing knowledge.

## Hintergrund

In den kommenden Jahren wird infolge des demografischen Wandels von einer Zunahme der zu betreuenden Menschen in der stationären Langzeitversorgung ausgegangen [1]. Vor diesem Hintergrund werden Sturzereignisse eine große Herausforderung sowohl für Pflegeheimbewohner:innen als auch für die Pflegefachkräfte in Langzeitpflegeeinrichtungen darstellen. Als ein Sturz wird ein unvorhergesehenes Ereignis beschrieben, welches dazu führt, dass eine Person unbeabsichtigt auf dem Boden oder einer anderen tieferen Ebene aufkommt [9].

Obwohl es in Deutschland keine systematische Erhebung zur Sturzhäufigkeit und zu den damit verbundenen Folgen innerhalb von Pflegeeinrichtungen gibt, belegen Studien, dass ältere Menschen in der stationären Langzeitpflege mit einer mittleren Selbstständigkeit in den Aktivitäten des täglichen Lebens ein erhöhtes Risiko für Stürze aufweisen [3, 7]. Diese Stürze lassen sich auf verschiedene Formen altersassoziierter Einschränkungen (kognitiv, visuell, Muskelschwäche oder Gleichgewichtsprobleme) zurückführen. So zeigte ein Review von Rubenstein und Josephson auf, dass Bewohner:innen von Langzeitpflegeeinrichtungen deutlich häufiger als in Wohngemeinschaften lebende ältere Menschen stürzen [13]. Die Sturzereignisse können sich gravierend auf die selbstbestimmte Lebensführung der Bewohner:innen auswirken und deren körperliche Funktionalität dauerhaft beeinträchtigen. Zudem kann die Angst vor einem erneuten Sturz die Mobilität sowie Alltagsaktivitäten einschränken [14]. Daher verfolgt die stationäre Altenhilfe die Ziele, Stürze weitestgehend zu vermeiden oder bei erfolgtem Sturz sofortige Unterstützungsleistungen zu bieten, Autonomie und Selbstständigkeit der älteren Menschen aufrechtzuerhalten sowie die Pflegenden zu entlasten. Dabei können innovative Pflegetechnologien wie beispielsweise Sturzsensoren unterstützend im täglichen Betreuungs- und Pflegealltag wirken.

Daher steht in diesem Beitrag die Implementation eines Sturzsensors (cogvisAI; Cogvis Software und Consulting GmbH, Wien, Österreich), welcher an der Decke des Bewohner:innenzimmers angebracht ist und über 3D-Smart-Sensoren Bewegungen sowie Sturzgefährdungen erfassen kann, im Vordergrund. Je nach Sturzgefährdungspotenzial der Bewohner:innen können individuelle Einstellungen wie beispielsweise Auslösungspunkte sowie Sensibilität (z. B. Alarmierung beim Aufsetzen oder Aufstehen der Bewohner:innen) in der Plattform des Systems vorgenommen werden, um Pflegefachkräfte bei einer potenziellen Gefahr über die Rufanlage oder eine Smartphone-App zu alarmieren [12]. Die Smartphone-App kann dabei Sturzpräventions- und Sturzerkennungsalarme unterscheiden. Da durch das installierte Sensorsystem eine kontinuierliche Überwachung erfolgt, ist eine Zustimmung der Bewohner:innen oder deren Angehörigen erforderlich. Die Übertragung von Informationen erfolgt ausschließlich in anonymisierter Form.

Da die Akzeptanz der Nutzenden ein wesentlicher Einflussfaktor für den erfolgreichen Einsatz oder das Scheitern neuer Technologien ist [6, 15, 16], wurden Informationen zu Erwartungen und Erfahrungen der Nutzer:innen, welche den Implementationsprozess widerspiegeln, erhoben.

## Methodisches Vorgehen

Für die vorliegende Studie wurde ein qualitatives Design gewählt, um die Perspektive der professionellen Pflege in Bezug auf die Erwartungen an und Erfahrungen mit der Sturzsensorik (hindernde und fördernde Einflussfaktoren der Implementation) zu erheben. Im Oktober 2022 wurden Interviews mit Beschäftigten aus 2 Einrichtungen der stationären Langzeitpflege in Konstanz durchgeführt, die vorab von den Einrichtungen selbst festgelegt wurden. Voraussetzung für die Rekrutierung war lediglich, dass die Interviewpartner:innen Erfahrungen im Umgang mit dem Sturzsensor während ihrer Beschäftigung in der jeweiligen Einrichtung gemacht haben sollten. In der ersten Einrichtung erfolgten 3 Einzelinterviews mit Pflegefachkräften [I1, I2; I3] und in der zweiten langzeitstationären Pflegeeinrichtung wurde ein Gruppeninterview mit 3 Pflegefachkräften [I5] durchgeführt. Darüber hinaus fand in beiden Einrichtungen jeweils ein Einzelinterview mit einer Person in Leitungsfunktion statt [I4; I6]. Alle interviewten Personen waren weiblich. Zwischen der Implementation der Sturzsensoren und den Interviews lag ein Zeitrahmen von einem bis 3 Monaten. Die Personen wurden jeweils mittels teilstrukturiertem Interviewleitfaden befragt (Zusatzmaterial online: „Teilstrukturierte Interviewleitfäden“). Hierbei wurden die Fragen offen gestaltet, um retrospektiv die Erwartungen sowie die Erfahrungen der Nutzer:innen erheben zu können. Die Interviews wurden in Besprechungsräumen in den jeweiligen Einrichtungen geführt, audiotechnisch aufgezeichnet und anschließend transkribiert. Die durchschnittliche Interviewdauer betrug 30 min.

Die inhaltliche Analyse wurde mit der Software MAXQDA 12 durchgeführt. Die Kategorienbildung erfolgte zunächst deduktiv auf Grundlage des Interviewleitfadens; anhand des Materials konnten weitere induktive Kategorien gebildet werden. Um die intersubjektive Nachvollziehbarkeit gemäß Gütekriterien qualitativer Forschung zu erzielen, erfolgte ein kontinuierlicher Austausch zwischen den beiden Personen, welche die Interviews durchführten und auswerteten.

## Ergebnisse

Die Analyse der Interviewdaten brachte 5 Hauptkategorien hervor: (1) Erwartungen an die Sturzsensorik, (2) fördernde Faktoren der Implementation, (3) hemmende Faktoren der Implementation, (4) Einsatz und Mehrwert für die ambulante pflegerische Versorgung sowie (5) Zukunftserwartungen und -wünsche.

### Erwartungen an die Sturzsensorik

Mittels der Interviews konnten die Erwartungen der professionell Pflegenden hinsichtlich der Sturzsensorik erhoben werden. Die Pflegefachkräfte nahmen an, dass sie *schneller auf einen Sturz oder eine Gefährdung hingewiesen werden* [I1, Z18]. Die professionell Pflegenden gingen von einem erhöhten subjektiven Sicherheitsgefühl der Pflegenden aus und erwarteten, dass die Versorgung weiterer Bewohner:innen fokussierter möglich sei [I2, Z12; I4, Z10]. Zusätzlich erhofften sich die professionell Pflegenden den Erhalt der Autonomie und Bewegungsfreiheit der Bewohner:innen [I2, Z12; I5, Z1]. Zusätzlich hatten die Nutzer:innen die Erwartung, dass sie durch die Abgabe von Verantwortung an eine assistive Technologie eine gewisse Entlastung erfahren [I2, Z10].

### Fördernde Faktoren der Implementation

Die Erkenntnisse deuten grundsätzlich auf positive Erfahrungen mit der Sturzsensorik hin. Als besonders fördernd sahen die Interviewten, dass zeitnahe Information über die Sturzgefährdung oder einen stattgefundenen Sturz erfolgten [I1, Z18]. Aufgrund des Potenzials zur Vermeidung von Stürzen ermöglichte die neue Technologie, die Autonomie sowie Bewegungsfreiheit älterer Menschen aufrechtzuerhalten, ohne eine stetige persönliche Überprüfung durch die Pflegefachkraft notwendig zu machen [I1, Z40; I4 Z12]. Das damit in Verbindung stehende subjektive Sicherheitsempfinden führte bei den Pflegefachkräften zu einem psychischen Entlastungseffekt, da nicht das Gefühl bestand, überall gleichzeitig sein zu müssen und die Versorgungssituation nicht durch Routinegänge unterbrechen zu müssen [I4, Z12].

Aus Sicht der professionell Pflegenden hatte die Sturzsensorik einen deutlichen Mehrwert im Vergleich zum vorher verwendeten Sturzerkennungssystem in Form einer Sturzmatte:

„Wir haben ja teilweise auch diese Sturzmatten, die funktionieren mal mehr, mal weniger. Wenn man da einmal kurz draufsteht, dann geht halt schnell der Alarm runter, und die sind halt so ein bisschen ja nicht so toll, sage ich jetzt mal.“ [I4, Z8]

Beispielsweise legten die befragten Personen dar, dass die Sturzsensorik geeignet sei, um Sturzgeschehnisse im gesamten Bewohner:innenzimmer und nicht nur vor dem Bett zu erkennen. Des Weiteren waren Fehlalarme durch heruntergefallene Gegenstände bei der Sturzsensorik nicht möglich. Bei der Mobilisation oder pflegerischen Versorgung der Bewohner:innen sei kein Wegräumen eines *Hindernisses* [I5, Z74] notwendig. Weiterhin wurden mobile Patient:innen durch die Sturzsensorik nicht in ihrer Bewegungsfreiheit eingeschränkt. Es gibt keine *Stolperfalle *[I5, Z29], welche direkt vor dem Bett liegt. Für Personen mit Hinlauftendenzen wurde die *Abwesenheitskontrolle* der Sturzsensorik [I4, Z10] positiv hervorgehoben. Durch die zuvor getätigten Einstellungen wurden die professionell Pflegenden bei Abwesenheit informiert und konnten rechtzeitig reagieren [I1, Z18].

Zudem verdeutlicht die folgende Aussage, dass durch die ausschließlich schematische Abbildung der Bewohner:innen die Privatsphäre und der Datenschutz gewährleistet werden:

„Datenschutzmäßig super, weil man sieht ja nicht die Person, man sieht ja wirklich nur diese Umrisse und wie sie sich dann auch im Zimmer bewegt.“ [I1, Z24]

Die Bedienoberfläche der Sturzsensorik wurde zum Zeitpunkt der Interviews ausschließlich von einer befragten Person genutzt, von dieser jedoch als *selbsterklärend und intuitiv *empfunden [I4, Z76]. Der zeitliche Aufwand der Implementierung im Vergleich zum Output wurde als gering angesehen:

„Also ich finde, vergleichsweise ist der Aufwand ziemlich gering gewesen. Das Aufwendigste war die Installation eigentlich, weil man Techniker halt einberufen musste (…).“ [I4, Z14]

Insgesamt wurde der technologische Reifegrad der Sturzsensorik als fortschrittlich beschrieben, und aus Sicht vieler professionellen Pflegekräfte wies die Technologie ein erhöhtes Potenzial auf, der Sturzgefährdung entgegenzuwirken [I4, Z76; I1, Z34; I5, Z42].

### Hemmende Faktoren der Implementation

Zudem wurden verschiedene hemmende Faktoren der Implementation benannt:

„Natürlich braucht es auch Kritik, weil sonst können wir ja nicht dran arbeiten, dass es weitergeht und besser wird und für uns noch leichter wird. Aber am Anfang sind natürlich viele Dinge erst mal ein bisschen (…) zeitaufwendig. Man muss natürlich gucken und das eingeben, man muss es im Team kommunizieren, und da ist immer, was ich echt ein Phänomen finde, dass die Jungen, die ja eigentlich mit Technik groß werden, dass die am wenigsten Toleranz für diese neuen Techniksachen haben.“ [I1, Z34]

Somit wurden hemmende Faktoren auf technischer, einrichtungsbezogener sowie individueller Ebene wahrgenommen. Als technikbezogener hemmender Faktor wurde von den Pflegefachkräften das Erfordernis zur individuellen – und zu Beginn recht zeitaufwendigen – Einrichtung der Sturzsensorikmodule für die einzelnen Bewohner:innen benannt. Des Weiteren wurden Fehlalarme als problematisch angesehen:

„Eben die Fehlalarme, teilweise Alarme, obwohl gar keiner im Zimmer war. (…) Es geht der Alarm aufs Handy los, und je nachdem, was gerade ist, müssen die dann losrennen. Und das ist natürlich in dem Moment dann eher, ja störend.“ [I6, Z18]

Der Aussage lässt sich entnehmen, dass die ausgelösten Fehlalarme den Arbeitsablauf ohne eine vorliegende Sturzgefährdung unterbrechen und diese als unnötige Belastung wahrgenommen wurden.

Ein weiterer wichtiger Aspekt ist, dass bei weiterführenden technischen Problemen, welche nicht selbstständig vor Ort gelöst werden können, eine Kontaktmöglichkeit über eine Hotline bestand. Nach einem gewissen Zeitraum erfolgte ein Rückruf, und eine Interviewpartnerin gab an, dass es wünschenswert sei, nicht auf diesen Rückruf warten zu müssen, sondern die Problemlösung direkt zu gewährleisten [I6, Z54].

Als einrichtungsbezogener hemmender Faktor der Implementation wurden die mangelnde Wissensvermittlung und Informationsbereitstellung im Umgang mit der Sturzsensorik beschrieben:

„Ich habe erst 3 Wochen später erfahren, wie ich das Ding überhaupt ausschalte, weil dann irgendjemand mal hochkam und gesagt hat: ‚Du, jedes Mal, wenn du reingehst, musst du es ausschalten.‘ Das wusste ich nicht. Man hat sich gewundert, warum ständig Alarm klingelt, wenn man das Zimmer betritt.“ [I3, Z16]

Die Aussage verdeutlicht, dass Schulungen und Informationen zu der neu eingesetzten Technologie essenziell sind, um einen fehlerhaften Umgang mit der Sturzsensorik zu verhindern. Außerdem hoben die professionell Pflegenden hervor, dass es zu Beginn weitere Schwierigkeiten gab. Beispielweise wurden die Sensoren von der Stromversorgung getrennt [I1, Z40; I2, Z14; I3, Z12; I4, Z40]. Der Grund war nicht eindeutig bekannt, aber es wurde von einem fehlenden Bewusstsein der Mitarbeitenden ausgegangen. Ein weiteres Problem bestand darin, dass nicht alle Pflegefachkräfte Zugang zur Bedienoberfläche der cogvisAI-Plattform hatten. Dies führte im Nachtdienst dazu, dass die zuvor festgelegten Auslösepunkte nicht geändert werden konnten. Dadurch war es nicht möglich, die gewünschte Neuanordnung des Mobiliars vorzunehmen, um Fehlalarme zu verhindern.

Von den Interviewten mit längerer Berufserfahrung wurde angemerkt, dass insbesondere jüngere Kolleg:innen eine desinteressierte bzw. ablehnende Haltung gegenüber der Technik hätten, da diese einen problemlosen Umgang mit dem System erwarten würden und sich nicht aktiv mit der Lösung potenziell auftretender technischer Probleme beschäftigen wollen [I1, Z34; I4, Z40].

### Einsatz und Mehrwert für die ambulante pflegerische Versorgung

Zum Zeitpunkt der Interviews wurde die Sturzsensorik ausschließlich in der stationären Langzeitversorgung als assistives Unterstützungssystem implementiert. Ein weiterer Interessenschwerpunkt der vorliegenden Studie war die Einstellung der Pflegefachkräfte hinsichtlich des Einsatzes und Mehrwerts der neuen Technologie für die ambulante pflegerische Versorgung. Bei den folgenden Aussagen ist zu berücksichtigen, dass die Interviewten im stationären Setting, welcher sich hinsichtlich der organisationalen Strukturen vom ambulanten Bereich unterscheidet, tätig sind.

Insgesamt schilderten die interviewten Personen, dass der Einsatz und Mehrwert abhängig von der Ausgangslage hinsichtlich des Gesundheitszustandes bzw. der Diagnosestellung der älteren Personen seien [I3, Z68; I2, Z46]. Als wichtige Grundvoraussetzung der Implementation im ambulanten Setting wurde die Gewährleistung der Privatsphäre der Nutzer:innen angesehen. Die Überwachungsmöglichkeit von An- bzw. Zugehörigen wurde als befremdlich und unerwünscht hervorgehoben [I1, Z64]. Eine selbstbestimmte Deaktivierung der Technologie wurde als Lösungsansatz zur Sicherung der Privatsphäre vorgeschlagen. Ein weiterer bedeutsamer Aspekt, der als hemmender Faktor angesprochen wurde, sind Fehlalarme. Diese wurden für das ambulante Setting als deutlich kritischer hervorgehoben als im stationären Bereich. Aus der Aussage einer Interviewten geht hervor, dass im ambulanten Setting deutlich mehr Zeit aufzuwenden wäre, um Fehlalarme zu verifizieren. Aufgrund der personellen Voraussetzungen im Pflegeberuf wäre dies aber kritisch zu betrachten [I3, Z50].

Als fördernder Faktor des Einsatzes wurde die zeitnahe Erkennung und damit verbundene Versorgung von Stürzen in der eigenen Häuslichkeit beschrieben [I1, Z68]. Zusätzlich wurde ein beruhigendes Gefühl für die An- und Zugehörigen durch den Sturzsensor unterstellt [I2, Z50].

### Zukunftserwartungen und -wünsche

Die Interviews liefern Erkenntnisse zu den einrichtungs-, technik- sowie systembezogenen Zukunftserwartungen und -wünschen an die Weiterentwicklung der Sturzsensorik. Grundsätzlich gaben die Pflegefachpersonen als einrichtungsbezogene Zukunftserwartung an, dass Schulungsangebote zur angemessenen Auseinandersetzung mit einem assistiven Unterstützungssystem zu gewährleisten seien [I2, Z18; I3, Z16; I4, Z60]. Die Schulungen sollten hierbei Aspekte wie Bedienungsfunktionen sowie Selbsterfahrungen enthalten. Die Bedienungsfunktionen wurden als ein Faktor benannt, weil die Notwendigkeit bestehe, die Sturzsensorik bei der Versorgung der Bewohner:innen aktiv auszuschalten und beim Verlassen des Raumes wieder zu aktivieren, um Fehlalarme zu vermeiden [I3, Z24]. Zusätzlich wurde der Selbsterfahrung ein hoher Stellenwert beigemessen. Zum Beispiel wurden eigene Erfahrungen zum Sturzerkennungsradius als dienlich angesehen, um ein höheres Sicherheitsgefühl zu entwickeln [I2, Z38]. Als weiterer Wunsch wurde das Zugriffsrecht auf die Bedienoberfläche benannt, um notwendige Anpassungen im System, aufgrund von Veränderungen der Patient:innensituation, selbstständig vornehmen zu können [I3, Z72].

Eine technikbezogene Zukunftserwartung an die Weiterentwicklung der Sturzsensorik war die Reduktion der derzeitig auftretenden Fehlalarme, damit die auszuführende Tätigkeit ohne Unterbrechung weiter fortgeführt werden könne [I6, Z26]. Zusätzlich wurde der Wunsch nach einer Veränderung angeführt, dass das Assistenzsystem mit dem Ruftaster kombiniert wird [I3, Z72].

Der systembezogene Zukunftswunsch bezieht sich auf eine finanzielle Beteiligung der Versicherungen, da aufgrund vermiedener Stürze und deren Folgen langfristig Kosten eingespart werden können:

„Da wäre es natürlich schon gut, wenn so was über die Krankenkasse dann schon auch mitfinanziert wird. (…) Auch für die Krankenkasse wäre es ja gut. Sie hätten ja nachher auch Vorteile, wenn weniger Stürze sind, weniger Oberschenkelhalsbruch und dadurch mehr wirklich Unfälle vermieden werden können.“ [I1, Z70]

## Diskussion

Obwohl Assistenzsysteme wie die Sturzsensorik eine hohe Relevanz aufweisen, wurde die Implementation von Sturzsensoren bislang wenig untersucht. Die Pflegefachkräfte erwarteten von einer solchen Technologie die Aufrechterhaltung von Autonomie der Bewohner:innen über die Vermeidung von Stürzen und Folgeschäden. Dies unterstreicht die erforderliche kritische Perspektive auf „smarte“ Technologien, die nicht nur hinsichtlich ihrer Usability, sondern auch in Bezug auf deren Effekte auf ältere Menschen betrachtet werden sollten [10], so wie es in Ausführungen zur Soziogerontechnologie vermehrt aufgegriffen wird [11]. Dies gilt auch, wenn ein solcher Sensor eher „passiv“ vorhanden ist als „aktiv“ genutzt wird.

Neben den Vorteilen für die Bewohner:innen erwarteten die Pflegefachkräfte eine (emotionale) Entlastung durch die Abgabe von Verantwortlichkeiten. Insgesamt verdeutlichen die Ergebnisse, dass keine Diskrepanzen zwischen den Erwartungen an und Erfahrungen mit der Technologie vorlagen, wie sie sich im Sinne der sozialen Praxis bei vielfältigen anderen Sensoren zeigen [5]. Die aufgezeigten positiven Assoziationen mit bzw. Erwartungen an den Sensor machen deutlich, dass die Technologie nicht nur als Arbeitserleichterung, sondern gar als „Heilsbringer“ imaginiert und konstruiert wird [2]. Dies erstaunt mit Blick auf die Fehlalarme: Obwohl solche Fehlalarme zu einer Beeinträchtigung der Arbeitsabläufe (nochmals verstärkt im ambulanten Bereich) – und somit längerfristig einer ggf. daraus resultierenden Ablehnung der digitalen Technologie – führen können [4], zeigt sich in dem Befragungssample eine vergleichsweise unhinterfragte Technikakzeptanz. Es scheint eine Legitimation der technischen Herausforderungen zu geben, welche sich darauf zurückführen lässt, dass der Sensor auf der einen Seite ein Sicherheitsgefühl bei den Pflegefachkräften selbst hervorruft und auf der anderen Seite als instrumentelles Hilfsmittel zur Förderung der Autonomie und Sicherheit der Bewohner:innen wahrgenommen wird. Der letztgenannte Aspekt kann vermeintlich eine Selbstverpflichtung bzw. moralischen Druck bei den Pflegefachkräften hervorrufen. Als Resultat dessen werden technische Schwierigkeiten zum Wohle der Bewohner:innen einfach akzeptiert. In einem solchen Fall wären die Pflegefachkräfte jedoch eher als „Pfleger:innen der Technik“ tätig. Aus diesem Grund sollte sich bei der Weiterentwicklung der Technologie darauf konzentriert werden, die Fehlalarme zu minimieren, um die Sicherheit der Bewohner:innen zu erhöhen, Arbeitsprozesse zu erleichtern und das Vertrauen in die Funktionalität der Sturzsensorik zu fördern.

Auf technikbezogener Ebene wird von den Pflegefachkräften und Einrichtungsleitungen die Zeitspanne, welche für die Implementation der Technologie aufgewendet werden muss, sowohl als hemmender als auch fördernder Faktor benannt. Diese Diskrepanz ist dadurch erklärbar, dass zwar die durch eine:n Techniker:in erfolgende Installation des Sensors keine zusätzliche Zeit der Pflegefachpersonen in Anspruch nimmt. Der zusätzliche zeitliche Aufwand entsteht für das Fachpersonal hingegen durch erforderliche Konfigurationen auf der Plattform des Systems. Insofern sind eine einfache Bedienbarkeit und Informationsvermittlung über die Technologie für alle Beschäftigten wichtige Bausteine, um einen fehlerhaften Umgang – welcher schnell zu einer Enttäuschung führen kann – vorzubeugen.

Zusätzlich weisen die Ergebnisse darauf hin, dass Pflegefachpersonen unterschiedlich bereit sind, sich mit der neuen Technologie zu befassen. In den beiden langzeitstationären Pflegeeinrichtungen wird jüngeren Pflegefachpersonen weniger Interesse an neuen Technologien zugesprochen. Dies steht im Einklang mit vorangegangenen Erkenntnissen, welche auf eine geringere Technikbegeisterung bei jüngeren im Vergleich zu älteren professionell Pflegenden hinweisen [8].

### Limitationen

Es ist zu berücksichtigen, dass die Auswahl der Interviewteilnehmerinnen von den Einrichtungen eigenständig vorgenommen wurde und die selektive Auswahl zu einer potenziellen Verzerrung führen kann. Darüber hinaus ist anzumerken, dass die Anzahl der interviewten Personen nicht repräsentativ ist und die Erkenntnisse ausschließlich einen vorläufigen Eindruck vermitteln können. Der Zeitraum zwischen der Implementierung und den Interviews betrug maximal 3 Monate. Dies ist einerseits positiv, da die Erwartungen und Erfahrungen aktuell sind. Andererseits befindet sich die Implementation der Sturzsensorik noch im Anfangsstadium, und die Technikerfahrungen der Nutzer:innen können sich im weiteren Verlauf verändern. Hinsichtlich der Erwartungen der Pflegenden ist zu beachten, dass eine retrospektive Datenerhebung erfolgte, wobei das Erinnerungsvermögen als eine Limitation betrachtet werden sollte. Da alle interviewten Personen weiblich waren, konnten keine geschlechterspezifischen Analysen vorgenommen werden. Des Weiteren ist die Sturzsensorik lediglich in 2 langzeitstationären Einrichtungen implementiert worden, sodass die Erkenntnisse nicht zwingend auf andere Einrichtungen oder das ambulante Setting übertragbar sind. Insgesamt betrachtet sind aufgrund der begrenzten Anzahl der Interviews weitere Untersuchungen wünschenswert, um die vorliegenden Erkenntnisse zu verifizieren und zu vertiefen.

## Fazit für die Praxis

- Neue digitale Technologien haben das Potenzial, nachteilige Folgen eines Sturzes zu verhindern, und können eine wertvolle Ergänzung zur Sturzprävention darstellen.
- Nutzer:innengruppen müssen adäquat geschult werden, und auch einrichtungsintern sind die Erfahrungen mit der Technologie sowie damit verbundene Änderungen in den Arbeitsprozessen in der Implementationsphase zu betrachten.
- Auf Basis des kontinuierlichen Austausches mit allen beteiligten Beschäftigungsgruppen können Verfahrensanweisungen (weiter-)entwickelt werden.

### Supplementary Information

„Teilstrukturierte Interviewleitfäden“
