# Supplementary material for: Implementation of fall sensors in long-term care: An interview study to identify promoting and inhibiting factors from the perspective of professional caregivers
Source: Z Gerontol Geriatr. 2023 Nov 8;57(4):290–5. [Article in German] doi: 10.1007/s00391-023-02255-3 (PMC11208179; doi:10.1007/s00391-023-02255-3)
Supplement: Supplementary file 1 — „Teilstrukturierte Interviewleitfäden“ [file 391_2023_2255_MOESM1_ESM.pdf]

Teilstrukturierte Interviewleitfäden

Teilstrukturierter Interviewleitfaden – Pflegefachkräfte

**Kategorien**	**Interviewfragen**
1. Erfahrungszeitraum mit der Sturzsensorik	1.1. Wie lange arbeiten Sie bereits mit dem Sturzsensor?
2. Erwartungen an die Sturzsensorik	2.1. Wenn Sie an die Zeit zurückblicken, bevor der Sturzsensor im Hause war: Welche Erwartungen hatten Sie daran?<br>• Auch auf Basis von vorangegangenen Herausforderungen in der frühzeitigen Sturzerkennung<br>• Inwieweit haben sich diese Erwartungen erfüllt?
3. Fördernde und hemmende Faktoren der Implementation	3.1. Welche Vor- und Nachteile erkennen Sie mittlerweile, nachdem der Sturzsensor schon einige Zeit im Einsatz ist? Können Sie dies an konkreten Beispielen deutlich machen?<br>• Aufwand: Belastung vs. Entlastung<br>• Funktionalität bzw. Genauigkeit der Prävention/Erkennung eines Sturzereignisses
4. Erfahrungen mit der Technologie	4.1. Haben Sie Erfahrungen mit der Nutzung ähnlicher Technologien? Wie beurteilen Sie den Sturzsensor im Vergleich?<br>4.2. Welche Probleme sind bislang in Bezug auf die Nutzung des Sturzsensors aufgekommen?
5. Nutzungskompetenzen	5.1. Sind aus Ihrer Sicht besondere Kompetenzen zur adäquaten Nutzung notwendig?
6. Rückmeldung von Angehörigen	6.1. Welche Rückmeldungen haben Sie ggf. von Angehörigen pflegebedürftiger Bewohner:innen zu dem Sturzsensor erhalten?
7. Einsatz und Mehrwert für die ambulante pflegerische Versorgung	7.1. Wenn Sie an das häusliche Umfeld denken: Könnte ihrer Meinung nach ein solcher Sturzsensor auch in der ambulanten pflegerischen Versorgung einen Mehrwert bieten?<br>• Nennen Sie gerne ein Szenario für die erfolgskritischen Faktoren.
8. Nutzungsakzeptanz und Zahlungsbereitschaft	8.1. Auf Basis der Erfahrungen, die Sie bislang mit dem Sensor in dieser Einrichtung gemacht haben: Wären Sie für sich selbst oder für einen Angehörigen bereit, einen solchen Sturzsensor zu nutzen? Warum oder warum nicht?<br>8.2. Wären Sie auch dazu bereit, einen Preis dafür zu bezahlen? Wenn ja, wie hoch wäre dieser?

Teilstrukturierter Interviewleitfaden – Einrichtungsleitung

**Kategorien**	**Interviewfragen**
1. Rahmeninformationen	1.1. Wie groß ist ihre Einrichtung? (Anzahl Mitarbeiter:innen/Pflegekräfte, Anzahl Betten/Pflegebedürftige)<br>1.2. Wie viele professionell Pflegende arbeiten in der Einrichtung mit dem Sturzsensor?<br>1.3. Inwiefern sind andere Berufsgruppen im Umgang mit dem Sensor vertraut?
2. Beweggründe der Implementation	2.1. Was waren die Beweggründe, den Sturzsensor in Ihrer Einrichtung zu nutzen?
3. Erwartungen an die Sturzsensorik	3.1. Wenn Sie an die Zeit zurückblicken, bevor der Sturzsensor im Hause war: Welche Erwartungen hatten Sie daran?<br>• Auch auf Basis von vorangegangenen Herausforderungen in der frühzeitigen Sturzerkennung<br>• Inwieweit haben sich diese Erwartungen erfüllt?<br>• Sollte der Sensor weitere Aufgaben oder Anforderungen erfüllen?
4. Fördernde und hemmende Faktoren der Implementation	4.1. Welche Vor- und Nachteile erkennen Sie mittlerweile, nachdem der Sturzsensor schon einige Zeit im Einsatz ist? Können Sie dies an konkreten Beispielen deutlich machen?<br>• Aufwand: Belastung vs. Entlastung<br>• Funktionalität bzw. Genauigkeit der Prävention/Erkennung eines Sturzereignisses
5. Erfahrungen mit der Implementation und Technologie	5.1. Wie bewerten Sie den Aufwand zur Installation und Implementierung der Sturzsensorik in Ihrer Einrichtung?<br>5.2. Haben Sie Erfahrungen mit der Nutzung ähnlicher Technologien? Wie beurteilen Sie den Sturzsensor im Vergleich?<br>5.3. Welche Probleme sind bislang in Bezug auf die Nutzung des Sturzsensors aufgekommen?
6. Nutzungskompetenzen und Schulungen	6.1. Sind aus Ihrer Sicht besondere Kompetenzen zur adäquaten Nutzung notwendig?<br>6.2. Inwieweit gab es diesbezüglich Schulung?
7. Veränderungen durch die Implementation	7.1. Wie haben sich die Arbeitsabläufe und -anforderungen von professionell Pflegenden durch die Implementierung des Sturzsensors verändert?
8. Rückmeldung von Angehörigen	8.1. Welche Rückmeldungen haben Sie ggf. von Angehörigen pflegebedürftiger Bewohner:innen zu dem Sturzsensor erhalten?

9. Einsatz und Mehrwert für die ambulante pflegerische Versorgung	9.1. Wenn Sie an das häusliche Umfeld denken: Könnte ihrer Meinung nach ein solcher Sturzsensor auch in der ambulanten pflegerischen Versorgung einen Mehrwert bieten?<br>• Nennen Sie gerne ein Szenario für die erfolgskritischen Faktoren.
10. Nutzungsakzeptanz und Zahlungsbereitschaft	10.1. Auf Basis der Erfahrungen, die Sie bislang mit dem Sensor in dieser Einrichtung gemacht haben: Wären Sie für sich selbst oder für einen Angehörigen bereit, einen solchen Sturzsensor zu nutzen? Warum oder warum nicht?<br>10.2. Wären Sie auch dazu bereit, einen Preis dafür zu bezahlen? Wenn ja, wie hoch wäre dieser?
